# Morquio Disease as a cause of atlanto-axial subluxation

**DOI:** 10.11604/pamj.2013.15.72.2872

**Published:** 2013-06-25

**Authors:** Mohammed Yassaad Oudrhiri, Saadia Aidi

**Affiliations:** 1 Neurosurgery department, Ibn Sina Hospital, Rabat; Morocco; 2 Neurology and Neuropsychology A department (El Alaoui Faris M), ONO Hospital, Rabat; Morocco

**Keywords:** Morquio Disease, atlanto-axial subluxation, limbs weakness, shoulder dislocation

## Image in medicine

A 12 years old boy, with no past medical history, presented to the neurology department with a 7 years clinical course of untreated limping, progressive kyphosis, repeated shoulder dislocation, and, recently, progressive limbs weakness. On examination, the patient was short trunked with fixed cervical hyperextension and thoracolumbar kyphosis. Also noted were genu valgum and ligamentous laxity. Neurological examination showed a spastic quadriparesis (3/5), exaggerated reflexes and bilateral Babinsky sign. Sensory function was normal, so as intelligence. Axial and appendicular X-ray radiographs demonstrated odontoid hypoplasia and possible occipito-cervical dislocation, a platyspondyly (image A, arrow), acetabular dysplasia (image B, arrow) and metacarpal irregular proximal ends (image C, arrow). MRI of the craniocervical junction revealed severe spinal cord compression at C1-C2 level with marked instability and dislocation (image D, arrow), odontoid hypoplasia associated with a soft tissue mass around (image E, arrow). All were suggestive of the mucopolysaccharidoses? origin. Laboratory investigations showed an over excretion of the keratane sulfate which comforted the diagnosis. The differential diagnosis of the image can be made with the atlanto-axial subluxation in rheumatoid arthritis, ankylosing spondylitis, and pseudo-gout (CPPD). The patient was proposed for a cervical posterior decompression with occiput-to C2 fusion, which was declined by his family when surgical procedure risks were explained.

**Figure 1:**
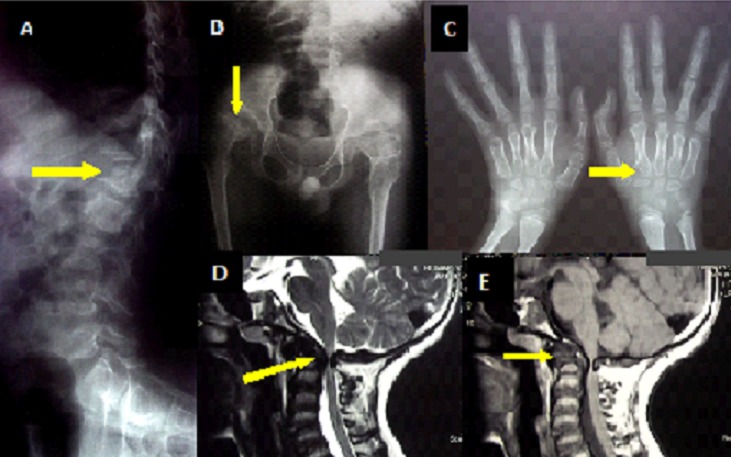
X-ray appendicular radiographs showing a platyspondyly (image A), acetabular dysplasia (image B) and metacarpal irregular proximal ends (image C). Images D and E, showing the marked atlanto-axial instability, and spinal cord compression

